# The Human Isoform of RNA Polymerase II Subunit hRPB11bα Specifically Interacts with Transcription Factor ATF4

**DOI:** 10.3390/ijms21010135

**Published:** 2019-12-24

**Authors:** Sergey A. Proshkin, Elena K. Shematorova, George V. Shpakovski

**Affiliations:** 1 Laboratory of Mechanisms of Gene Expression, Shemyakin-Ovchinnikov Institute of Bioorganic Chemistry, Russian Academy of Sciences, 117997 Moscow, Russia; sproshkin@gmail.com (S.A.P.); elenashe@mail.ru (E.K.S.); 2 Engelhardt Institute of Molecular Biology, Center for Precision Genome Editing and Genetic Technologies for Biomedicine, 119991 Moscow, Russia

**Keywords:** RNA polymerase II, human isoforms of Rpb11, hRPB11bα, hRPB11a, ATF4, yeast two-hybrid system

## Abstract

Rpb11 subunit of RNA polymerase II of Eukaryotes is related to N-terminal domain of eubacterial α subunit and forms a complex with Rpb3 subunit analogous to prokaryotic α_2_ homodimer, which is involved in RNA polymerase assembly and promoter recognition. In humans, a *POLR2J* gene family has been identified that potentially encodes several hRPB11 proteins differing mainly in their short C-terminal regions. The functions of the different human specific isoforms are still mainly unknown. To further characterize the minor human specific isoform of RNA polymerase II subunit hRPB11bα, the only one from hRPB11 (POLR2J) homologues that can replace its yeast counterpart in vivo, we used it as bait in a yeast two-hybrid screening of a human fetal brain cDNA library. By this analysis and subsequent co-purification assay in vitro, we identified transcription factor ATF4 as a prominent partner of the minor RNA polymerase II (RNAP II) subunit hRPB11bα. We demonstrated that the hRPB11bα interacts with leucine b-Zip domain located on the C-terminal part of ATF4. Overexpression of ATF4 activated the reporter more than 10-fold whereas co-transfection of hRPB11bα resulted in a 2.5-fold enhancement of ATF4 activation. Our data indicate that the mode of interaction of human RNAP II main (containing major for of hRPB11 subunit) and minor (containing hRPB11bα isoform of POLR2J subunit) transcription enzymes with ATF4 is certainly different in the two complexes involving hRPB3–ATF4 (not hRPB11a–ATF4) and hRpb11bα–ATF4 platforms in the first and the second case, respectively. The interaction of hRPB11bα and ATF4 appears to be necessary for the activation of RNA polymerase II containing the minor isoform of the hRPB11 subunit (POLR2J) on gene promoters regulated by this transcription factor. ATF4 activates transcription by directly contacting RNA polymerase II in the region of the heterodimer of α-like subunits (Rpb3–Rpb11) without involving a Mediator, which provides fast and highly effective activation of transcription of the desired genes. In RNA polymerase II of *Homo sapiens* that contains plural isoforms of the subunit hRPB11 (POLR2J), the strength of the hRPB11–ATF4 interaction appeared to be isoform-specific, providing the first functional distinction between the previously discovered human forms of the Rpb11 subunit.

## 1. Introduction

All messenger RNAs in eukaryotic cell are synthesized by RNA polymerase II (RNAPII), a complex enzyme, which consists of 12 subunits of the total weight over 0.8 MDa. During transcription, RNAPII is a target of transient assembly of numerous transcription factors, such as the general transcription factors, coactivators (e.g., mediator), elongation factors, proteins for RNA processing and transcription termination.

Until recently, RNAPII has been mostly investigated in yeast *Saccharomyces cerevisiae* and huge breakthrough progress was made in the past two decades by resolving 3D structures of yeast RNAPII as well as of its complexes with several factors [1,2,3,4,5,6,7,8,9,10,11,12]. The overall structure of human RNAPII appears to be similar to the yeast one [1,2,3] as expected by the sequence conservation and the fact that a number of human subunits can functionally replace their yeast counterparts [13,14,15]. This was recently confirmed by 3D structure of mammalian (bovine) RNAPII with 3.4 Å resolution obtained by cryoelectron microscopy [6]. Despite high sequence and structure similarity to yeast RNAPII, there are likely unique features associated with the increased complexity of human RNAPII transcription system.

All 12 subunits of RNAPII are closely homologous or identical to the subunits of nuclear RNA polymerase I and III, five of which are related to the core subunits of bacterial RNA polymerase [2,3]. Rpb11 subunit of RNAP II is related to the N-terminal domain of the eubacterial α subunit and forms a complex with the Rpb3 subunit analogous to the α_2_ homodimer, which is involved in RNA polymerase assembly and promoter recognition [16]. Together with Rpb2 subunit of RNAPII Rpb3–Rpb11 heterodimer forms a subcomplex, which corresponds to βα_2_ assembly intermediate of prokaryotic RNA polymerase [16,17]. Rpb3–Rpb11 plays a central role in the interaction of RNAPII with Mediator [18,19]. Consistent with this, mutations in the region of Rpb3, corresponding to activation target of bacterial α subunit, have been found to affect activator-dependent, but not basal transcription [17].

In humans, a gene family has been identified that potentially encodes several hRPB11 (POLR2J) proteins differing mainly in their C-terminal regions [20,21,22,23]. The *POLR2J1 (RPB11a)* gene encodes hRPB11a subunit representing the major component of mammalian RNAPII complex. Other members of the family, the *POLR2J2 (RPB11b)* and *POLR2J3 (RPB11c)* genes, yield several minor mRNAs resulting from alternative splicing [20,21]. Whether the transcripts of the *POLR2J2 (RPB11b)* gene are translated remain to be established. Remarkably, using complementation assay, it was demonstrated that only minor isoform hRPB11bα (the product of expression of the *POLR2J2* gene) is functional in yeast whereas the major (the true orthologue of Rpb11 from all other species) hRPB11a is not [20]. Obviously, the existence of a variety of human-specific subunit isoforms indicates the fact that the more intricate mechanisms for regulation of transcription in human cells have evolved [24,25,26]. The functions of the different minor isoforms are currently unknown and need to be investigated.

Here, we provide the first evidence about in vivo functional distinction between the previously discovered [20,21,22,23,24,25,26] distinct human isoforms of the hRPB11 (POLR2J) subunit of RNA polymerase II of *Homo sapiens*: The strength of the hRPB11–ATF4 interaction in corresponding transcription complexes appeared to be isoform-specific.

## 2. Results

Previously, we have shown that, in drastic difference with all other species, small indispensable subunit Rpb11 of RNA polymerase II is encoded in *Homo sapiens* by four different genes located on chromosome 7 [20,21]. One gene, *POLR2J1* (~5.5 kb long), is orthologous to the corresponding *rpb11* genes in other species and encodes the usual (trivial), main isoform of hRPB11 (POLR2J) subunit called hRPB11a (117 aa long) which, as judged by its evolutionary history, is the subject of strong purifying selection because is strictly conserved in all warm-blooded animals.

Among other *POLR2J* genes of *Homo sapiens* (*POLR2J2*–*POLR2J4*), which are much longer (approximately 34.5 kb in length each), two, *POLR2J2* and *POLR2J3*, not only have preserved all the hRPB11 (POLR2J) subunit coding potential, but can even produce plural variants of this subunit, including the most interesting isoforms hRPB11bα (115 aa; EMBL Acc.: AJ277739, Protein ID: CAC18329; CCDS 43627) and hRPB11bβ (116 aa; EMBL Acc.: AJ277740, Protein ID: CAC18330) [20,21]. The most similar of the two subunits, hRPB11a is the minor isoform hRPB11bα, which differs from the aforementioned hRPB11a (the major isoform) by the absence in its N-terminal part of one of the two consecutive lysine residues (Lys-17 or Lys-18) on the border of the first and second exons, and by a completely different sequence (10 instead of 11 amino acids) at the very C-terminal end of their primary structures, encoded by the completely different fourth exon [20,21].

To characterize the hRPB11bα isoform and to discover unique functional features, which can distinguish it from the major isoform hRPB11a, we used it as bait in a yeast two-hybrid screening of a human fetal brain cDNA library and identified transcription factor ATF4, a member of the ATF/CREB (activating transcription factor/cAMP response element binding protein) family of basic region-leucine zipper proteins [27,28,29], as one of the most prominent partners of this unique human protein.

### 2.1. Yeast Two-Hybrid Screening

**Figure 1:**
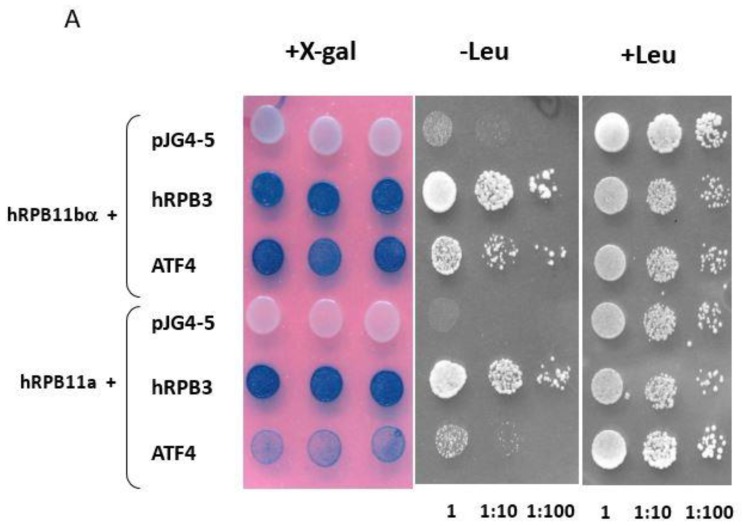

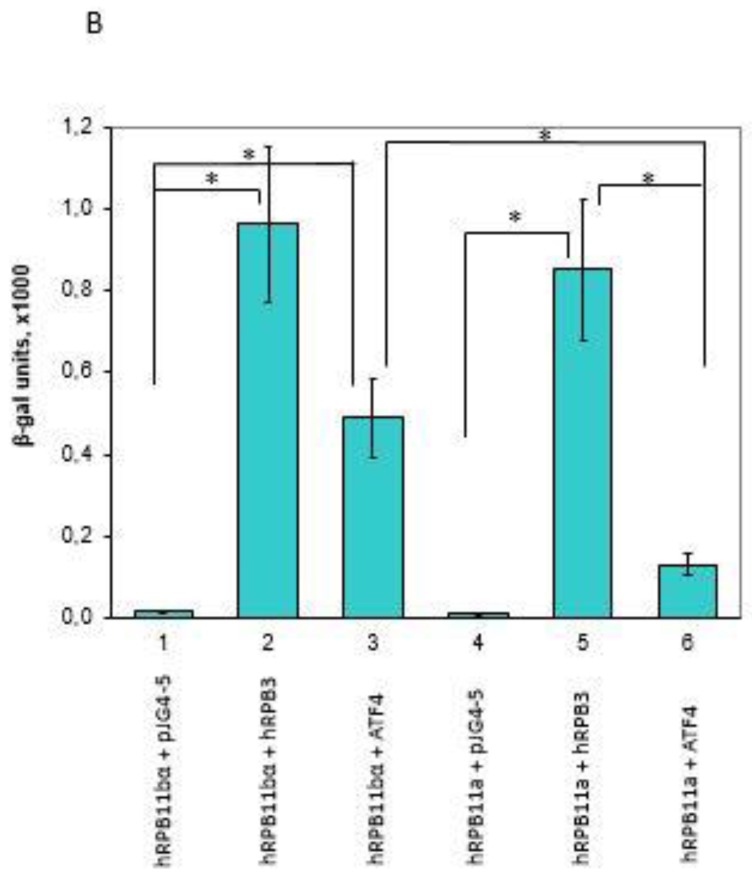
(**A**) Two-hybrid analysis of hRPB11bα interactions. Serial dilutions of SKY191 yeast cells containing the indicated plasmid combinations were spotted onto SD-Gal/Raff plates lacking triptophan, histidine, and uracil (-WHU) to verify that yeast contains both the bait and prey plasmids (left panel) or triptophan, histidine, uracil, and leucine (-WHUL) for demonstration of the interaction between the bait and prey (central panel). Three independent clones of each of the SKY191 transformants were spotted onto X-gal containing plate (right panel) for confirmation of positive interactions (the picture was made on the red background for the better visualization of both negative and positive clones). In comparison, the yeast strains containing an empty vector pJG4-5 or library-derived plasmid encoding C-terminal part of hRPB3 were used as negative or positive controls, respectively. (**B**) Assessment of interaction of hRPB11bα and ATF4. SKY191 yeast cells were cotransformed with the indicated constructs and assayed for β-galactosidase activity in the liquid medium by means of the Miller method [31]. The pairs of hRPB11bα–hRPB3 and hRPB11a–hRPB3 interacting proteins were taken as a positive control. Error bars: Standard deviation; statistical analysis: * *p* < 0.05 for all measurements.

To identify specific hRPB11bα-interacting proteins, we used the yeast two-hybrid system Interaction Trap [30]. A cDNA fragment encoding the full-length hRPB11bα was cloned in frame with the LexA DNA binding domain and was used as bait. Earlier, it was demonstrated that *hRPB11bα (POLR2J2)* mRNA was most abundant in the brain [20], so we screened a human fetal brain cDNA library. That library is based on the expression vector, which utilizes the galactose-inducible *GAL1* promoter to express library clones as fusions to a transcriptional activation domain B42. Twenty-five positive clones capable of simultaneously activating both reporters *LEU2* and *lacZ* in a galactose-specific manner were obtained and further characterized. Five of them contained the plasmids encoding the C-terminal part of RNA polymerase II subunit hRPB3, thus confirming the specificity of the screening. Four positive clones encoded the C-terminal part of transcription factor ATF4 (aa 221–351) (Figure 1). To define the specificity of interaction of hRPB11bα with ATF4, we tested the major isoform hRPB11a as bait in the same two-hybrid system. The intensity of interaction of ATF4 with hRPB11a was lower than that with the hRPB11bα, indicating selectivity of ATF4 binding to the two different human isoforms, whereas interaction with the C-terminal part of hRPB3 was independent of the isoforms tested (Figure 1B).

### 2.2. hRPB11bα Specifically Interact with ATF4 In Vitro

To confirm the in vivo interaction found by yeast two-hybrid system, we performed in vitro binding assay. We constructed a new vector pEXP2 to co-express interacting proteins in *E. coli*. The recombinant vector carries an M15 replication origin, the kanamycin resistance gene, the *LacI* gene, and the cloning expression region of pET15b (construction details will be described elsewhere). This vector can be used for co-expression together with any available ColE1-containing vector with a resistance selection marker other than kanamycin.

**Figure 2:**
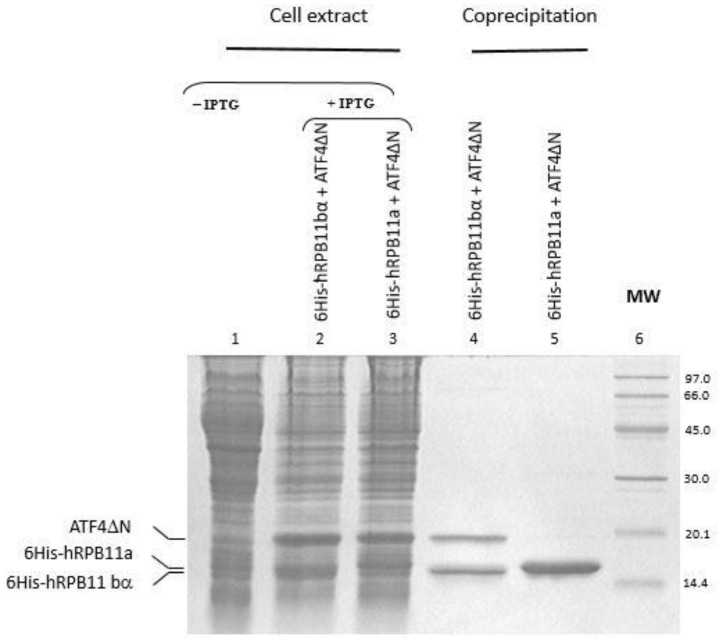
Co-precipitation of hRPB11bα and hRPB11a isoforms of RNA polymerase II subunit POLR2J (hRPB11) with ATF4 after their heterologous expression in *E. coli* cells (in vitro interaction of hRPB11bα and ATF4 proteins). Fifteen percent SDS-PAGE analysis of the proteins retained on TALON agarose. Recombinant proteins 6His-hRPB11bα (lane 4) and 6His-hRPB11a (lane 5) were immobilized on TALON agarose. Untagged ATF4ΔN protein retains on the agarose if it binds immobilized proteins. MW, molecular weight marker. The gel was visualized by Coomassie Blue R250 staining as described in Materials and Methods.

Full-length cDNAs of isoforms hRPB11a and hRPB11bα were cloned into pEXP2 as fusions with six contiguous histidines at their N-terminal. Full-length cDNA of ATF4 or its C-terminal part (221–351 aa) were cloned in pET21d and expressed as untagged proteins. We co-transformed plasmids encoding hRPB11a and hRPB11bα with the plasmids harboring cDNA of ATF4 and co-expressed the recombinant proteins in the same bacterial cell. The soluble supernatants were incubated with the Co^2+^-based TALON agarose, the beads were washed, and the absorbed proteins were eluted with imidazole. ATF4 as well as its C-terminal part were efficiently recovered with hRPB11bα, although they were not co-purified with hRPB11a (Figure 2). Due to lower affinity of ATF4 to hRPB11a, the transcription factor obviously easily dissociates during purification. This result confirms the obtained yeast two-hybrid (YTH) data and indicates specific interaction of the minor isoform hRPB11bα and ATF4.

hRPB11a differs from the minor isoform hRPB11bα in its C-terminal region, encoding by the fourth exon, and in the additional lysine residue (Lys-17) in the N-terminal part [20,21]. To delineate specific ATF4-interacting domains of hRPB11bα we constructed the deletion mutant of the minor isoform as a fusion with six histidines lacking all C-terminal sequence that differs from hRPB11a, i.e., the last 10 amino acids (aa 106–115). We used this construct in the co-purification assay as described above for the full-length proteins and found that such a deletion variant dramatically decreased its affinity to ATF4.

**Figure 3:**
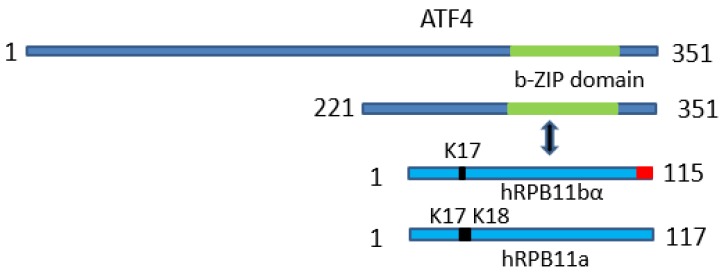
Schematic representation of the uncovered protein-protein interaction: The minor subunit of *Homo sapiens* RNA polymerase II hRPB11bα specifically interacts with transcription factor ATF4 through its C-terminal b-Zip domain.

In summary, as is schematically represented on Figure 3, our data indicate that minor subunit hRPB11bα of *Homo sapiens* RNA polymerase II interacts with transcription factor ATF4 through its C-terminal b-Zip domain and that the integrity of the hRPB11bα isoform protein structure is important for productive binding.

### 2.3. RPB11bα Enhances ATF4-Mediated Transactivation

**Figure 4:**
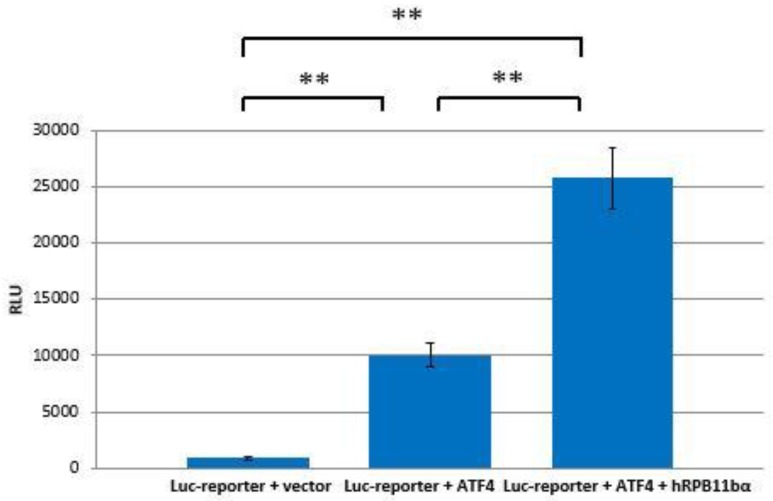
hRPB11bα is involved in ATF4-mediated transcription. HeLa cells were transiently transfected with 0.2 µg of the 3×ATF4-Luc reporter and 1 µg of the indicated expression vectors. Intensity of the light (560 nm) emitted as a result of the luciferase catalyzed chemiluminescent reaction is represented along the ordinate axis in relative light units (RLU = light emission/second). The data are presented as mean ± SD and representative of three independent experiments (** *p* < 0.005 for both measurements) performed in duplicate after normalization for transfection efficiency.

The interaction of hRPB11bα and ATF4 can be directly involved in activation of RNA polymerase II. To test this hypothesis, ATF4 and hRPB11bα cloned in mammalian expression plasmids and a luciferase reporter containing tandem ATF4 binding sites were cotransfected into HeLa cells. As shown in Figure 4, overexpression of ATF4 activated the reporter more than 10-fold whereas cotransfection of hRPB11bα resulted in two and a half fold (2.5×) enhancement of ATF4 activation.

## 3. Discussion

Eukaryotic RNA polymerase II subunit Rpb11 is an essential component of the transcriptional machinery that forms a heterodimer with Rpb3 subunit. This heterodimer plays an important structural role in the formation of the RNA polymerase II complex [16]. It was shown that bacterial α subunits (homologues of Rpb3 and Rpb11) are involved in transcription activation [32]. It was previously described that human Rpb3 (hRPB3) binds to transcription factors ATF4 [33] and myogenin [34], and that human Rpb11 (hRPB11a) interacts with Che-1 (AATF) [35]. These interactions lead to transcription activation without significant Mediator involvement [36], because the Mediator also binds the RNA polymerase II on the side of the Rpb3-Rpb11 heterodimer [37,38,39,40].

In 2001, in collaboration with French scientists, we first established that one of the subunits of human nuclear RNA polymerase II is encoded by at least several genes: Three isoforms of human hRPB11 (POLR2J) subunit, synthesized by the expression of two different genes: *POLR2J* and *POLR2J2*, were characterized in in vitro experiments, hRPB11a (basic, classical form), hRPB11bα, and hRPB11bβ [20]. Further, we identified four independent genes encoding different variants of hRPB11 RNA polymerase II subunit of *Homo sapiens* as part of human chromosome 7 [21]. Three genes, named *POLR2J1*, *POLR2J2*, and *POLR2J3*, are located as a single cluster with a total length of 214,530 bp in the genetic region 7q22.1 on the long arm of the chromosome; another gene, *POLR2J4* (31,040 bp), is located in the cytogenetic locus 7p13 of the short arm of chromosome 7. It was also shown that the expression of four human *POLR2J* genes (*POLR2J1*–*POLR2J4*) could lead to the synthesis of at least 14 species of mature mRNAs encoding slightly different isoforms of the hRPB11 subunit, 12 of which were detected by us as full-sized copies or clearly correlated cDNAs fragments in the available EST (expressed sequence tags) and cDNA databases [21].

In order to study the function of the most interesting isoforms hRPB11bα and hRPB11bβ, for the first time, we discovered *Homo sapiens* proteins interacting with these human-specific isoforms using genetic and biochemical approaches [25,26]. The functional characteristics of the detected partner proteins of hRPB11bα and hRPB11bβ isoforms indicate that these isoforms, like the main subunit of RNA polymerase II hRPB11a (POLR2J), are components of special transcription complexes and participate not only in the transcription of certain DNA matrices, but are also involved in the later stages of mRNA biogenesis [25,26]. Indeed, in addition to the components of the transcription complex of RNA polymerase II, such as hRPB3 and hRPB6, among the detected partner proteins were subparticles of other complexes involved in the later stages of human mRNA biogenesis, in particular a number of the subunits of the translation initiation factor hEIF3 [25].

Here, in this work, we analyzed in detail the specificity of interaction of major RNA polymerase II subunit hRPB11a (GenBank X98433, UniProt P52435, CCDS 5724, Ensembl-Tr: ENST00000292614.9 [transcript: POLR2J-201], protein–ENSP00000292614) and human-specific isoform hRPB11bα (GenBank AJ277739, UniProt Q9GZM3, CCDS 43627, Ensembl-Tr: ENST00000333432 [transcript: POLR2J2-201], protein–ENSP00000330898) with the b-Zip transcription factor ATF4, which was identified as one of the most prominent interactor of these α-like subunits under study of the human RNA polymerase II.

We have established that ATF4 through its C-terminal b-Zip domain interacts with one of the human specific isoforms of Rpb11, i.e., the minor subunit hRPB11bα, in the yeast two-hybrid system, and this interaction was also confirmed by coprecipitation of the proteins in vitro after their heterologous expression in a bacterial system. Overexpression of ATF4 and hRPB11bα in the human HeLa cell line increases transcription levels in 12.5 times (in luciferase test).

We found that the intensity of interaction of ATF4 with hRPB11a was lower than that with hRPB11bα, indicating selectivity of ATF4 binding to the two different human isoforms. Previously, it was shown that another α-like subunit of RNA polymerase II, hRPB3, directly binds ATF4 in vivo and in vitro and enhances ATF4 transactivating activity, indicating that this interaction (hRPB3–ATF4) provides the main platform for ATF4 activation of the major form of human (and all other mammalian) RNA polymerase II enzyme [33]. We demonstrated the interaction of hRPB11bα with the C-terminal part (aa 221–351) of ATF4 (Figure 3). The strength of the interaction appeared to be isoform-specific, giving the first functional distinction between the human forms of the hRPB11 subunit.

ATF4 is a transcription factor activated during stress conditions. Thus, the ATF4–hRPB11bα interaction probably is necessary for rapid gene activation in cellular response to different stress signals. Indeed, mRNA of hRPB11bα is found in polysomes after treatment of cells with 5′-fluorouracil and may be implicated in the resistance formation to this anticancer drug [41].

## 4. Materials and Methods

### 4.1. Yeast Two-Hybrid Screen

For two-hybrid selection, the complete open reading frame of human isoform hRPB11bα was cloned into bait vector pMW103 [42] in frame with the LexA DNA binding domain as *Eco*RI/*Bam*HI insert. pMW103-hRPB11bα was introduced along with the *LacZ* reporter plasmid pDR8 (Invitrogen) into the *S. cerevisiae* strain SKY 191 (MATα, *trp1*, *his3*, *ura3*, *2lexAop-LEU2*, *3cIop-Lys2*) by standard lithium acetate transformation [43]. Cells were plated on minimal synthetic defined medium (SD-Glu): 2% glucose (Glu), 0.67% yeast nitrogen base, supplemented with the required bases and amino acids, lacking histidine, and uracil (-HU). In the SKY 191, the upstream regulatory elements of the chromosomal *LEU2* gene are replaced by two copies of the LexA operator and the pDR8 contains the *LacZ* gene also under the control of 6 *LexA* operators. Absence of transcriptional activation capability of the *LEU2* and *LacZ* reporters by the baits in the SKY 191 was verified prior to library screening. Human fetal brain cDNA library (Invitrogen) based on the expression vector pJG4-5, which utilizes the galactose-inducible *GAL1* promoter to express library clones as fusions to a transcriptional activation domain (the “acid blob”, B42), was directly transformed into yeast containing pMW103-hRPB11bα and pDR8. Cells were plated on each of 15 SD-Glu plates (240 × 240 mm) lacking triptophan, histidine and uracil (-WHU) and allowed to grow for 3 days at 30 °C. In the next step, a homogenized slurry was prepared from the pool of primary transformants (approximately 3 × 10^5^ colonies), aliquoted, frozen, and stored at −70 °C. Then, an aliquot was thawed, diluted with SD-Gal/Raff/-WHU (2% galactose, 1% raffinose instead of glucose) liquid medium, and incubated with shaking at 30 °C for 5 h. About 10^6^ cells were plated on each of 5 of 100 mm Gal/Raff/-WHUL plates (lacking leucine) and incubated for 5 days at 30 °C. Positive clones, which were able to grow on galactose/raffinose media lacking leucine, were tested for galactose-inducible transcriptional activation of *lexAop-lacZ* reporter by plating on 5-bromo-4-chloro-3-indoyl-d-galactoside (X-gal) containing medium. Plasmids from positive yeast clones were isolated, electroporated into *E. coli* XL-blue cells, and plated on media containing 50 µg/mL ampicillin. The recovered library-derived plasmids were further analyzed as positive candidates. To investigate the specificity of interaction of the hRPB11bα with the prey, cDNA of hRPB11a was cloned into pMW103. Resulting plasmid pMW103-hRPB11a was transformed into SKY 191 as described above along with pDB20 and library-derived plasmid.

### 4.2. Liquid ß-Galactosidase Assay

Exponentially growing cells in 2 mL SD Gal-Raff/−Ura –His −Trp media were collected by centrifugation, washed with Z-buffer (60 mM Na_2_HPO_4_, 40 mM NaH_2_PO_4_, 10 mM KCl, 1 mM MgSO_4_, pH 7.0), and resuspended in 2 mL of Z-buffer. Then, 200 μL of cells suspension were mixed with 800 μL of Z-buffer (1:5 dilution) and the optical density at 600 nm (OD_600_) was measured. Then, 20 μL of 0.1% SDS, 40 μL of chloroform were added, and tubes were vortexed for 10 s. The permeabilized cells were equilibrated to 30 °C for 15 min, then 200 μL of 4 mg/mL ONPG (o-nitrophenyl-β-d-galactoside) solution was added, and the reaction proceeded at 30 °C until a yellow color was observed. After the addition of Na_2_CO_3_ and centrifugation, the formation of o-nitrophenol was determined by measuring the optical density at 420 nm (OD_420_). β-galactosidase activity was calculated with the following equation: u = 1000 × OD_420_/(t × OD_600_ × 5).

### 4.3. Proteins Coexpression and Purification

Full-length cDNA of isoforms hRPB11a and hRPB11bα were cloned into pEXP2 as *Nde*I/*Bam*HI insert. The recombinant vector pEXP2 carries an M15 replication origin, the kanamycin resistance gene, the *LacI* gene, and the cloning expression region of pET15b. cDNA coding for C-terminal domain ATF4 (amino acids 221–351), isolated by two-hybrid selection, was cloned into the *Bam*HI-*Hind*III sites of pET21d. Full-length cDNA of ATF4 was amplified from the plasmid pCGN-ATF4 kindly provided by Dr. Tsonwin Hai (Ohio State University, Columbus, OH, USA) and cloned into pET21d as *Bam*HI-*Xho*I insert. To construct deletion variant of hRPB11bα without the fourth exon, the corresponding part of cDNA was amplified with the pair of specific primers (the structures of all oligonucleotides used in this work are available upon request) and cloned into the *Nde*I-*Bam*HI sites of pEXP2. Plasmids were transformed into *E. coli* BL21(DE3) strain. For co-expression, the two pairs of plasmids were transformed into the same strain. The cells were grown at 37 °C in LB media until the A_600_ 0.5. Then, IPTG was added to final concentration of 1 mM, and bacterial culture was incubated at 30 °C or at room temperature for 4 h or overnight, respectively. The cells were centrifuged, resuspended in TG500 (50 mM Tris-HCl, pH7.5, 5% glycerol, 500 mM NaCl, 1 mM β-mercaptoethanol) buffer, and lysed by sonication. The insoluble materials were removed by centrifugation. Next, TG500-equilibrated Talon Co^2+^ affinity bead suspension (Clontech Laboratories, Mountain View, CA, USA) was added to the lysate and the suspension was incubated for 1 h at 4 °C with slight agitation. Then, the beads were washed five times with TG500. Absorbed proteins were batch-eluted with TGI buffer (50 mM Tris-HCl, pH7.5, 5% glycerol, 250 mM imidazole, 100 mM NaCl, 1 mM β-mercaptoethanol) and analyzed by electrophoresis in 15% SDS polyacrylamide gels. The gels were visualized by Coomassie Blue R250 staining essentially as described in [44].

### 4.4. Cell Culture and Transfections

Human HeLa cells were grown in Dulbecco’s modified Eagle’s medium (DMEM) supplemented with 10% fetal calf serum. Transient transfections were performed using Lipofectamine 2000 reagent (Thermo Fisher Scientific, Waltham, MA, USA) according to the manufacturer’s instructions.

### 4.5. Plasmids

The reporter pSp1ATF-Luc containing one SP1 and three ATF binding sites (3×ATF4) downstream from SP1 and pCGN-ATF4 plasmids were kindly provided by Dr. Tsonwin Hai (Ohio State University).

### 4.6. Luciferase Assay

Luciferase assay was performed using Luciferase Assay Kit (Agilent Technologies, Inc., St. Clara, CA, USA) according to the manufacturer’s instructions.

## 5. Conclusions

The functional distinction between the previously discovered original isoforms of the hRPB11 (POLR2J) subunit of RNA polymerase II of *Homo sapiens* has been shown for the first time. We have demonstrated that minor hRPB11bα isoform of human RNA polymerase II specifically interacts with C-terminal b-Zip domain of ATF4, providing novel platform for binding of this transcription factor to heterodimeric region of the α-like subunits (Rpb3–Rpb11) in RNAPII complexes containing hRPB11bα isoform of POLR2J subunit.

## Abbreviations

ATF/CREB	activating transcription factor/cAMP response element binding protein
b-Zip	basic region-leucine zipper domain in ATF/CREB family of proteins
EST	expressed sequence tag
hEIF3	13-subunits factor initiation of transcription #3 of *Homo sapiens* (human eukaryotic initiation factor 3)
RLU	relative light units
RNAPII	RNA polymerase II
YTH	yeast two-hybrid system
